# Maintenance of a central high frequency synapse in the absence of synaptic activity

**DOI:** 10.3389/fncel.2024.1404206

**Published:** 2024-11-18

**Authors:** Sascha Lessle, Lena Ebbers, Yvette Dörflinger, Simone Hoppe, Michaela Kaiser, Hans Gerd Nothwang, Christoph Körber

**Affiliations:** ^1^Department of Functional Neuroanatomy, Institute of Anatomy and Cell Biology, Heidelberg University, Heidelberg, Germany; ^2^Division of Neurogenetics, Center of Excellence Hearing4All, School of Medicine and Health Sciences, Carl von Ossietzky University Oldenburg, Oldenburg, Germany; ^3^Research Center for Neurosensory Science, Carl von Ossietzky University Oldenburg, Oldenburg, Germany

**Keywords:** calyx of Held, tetanus toxin, VCN, MNTB, Math5

## Abstract

Activity has long been considered essential for circuit formation and maintenance. This view has recently been challenged by proper synaptogenesis and only mildly affected synapse maintenance in the absence of synaptic activity in forebrain neurons. Here, we investigated whether synaptic activity is necessary for the development and maintenance of the calyx of Held synapse. This giant synapse located in the auditory brainstem is highly specialized to maintain high frequency, high-fidelity synaptic transmission for prolonged times and thus shows particularly high synaptic activity. We expressed the protease tetanus toxin light chain (TeNT) exclusively in bushy cells of the ventral cochlear nucleus (VCN) of juvenile mice. Since globular bushy cells give rise to the calyx of Held, expression of TeNT in these cells specifically abolished synaptic transmission at the calyx without impairing general functionality of the central auditory system. Calyces lacked synaptic activity after two weeks of TeNT expression. However, this did not lead to major changes in presynaptic morphology, the number of active zones (AZs) or the composition of postsynaptic AMPA-type glutamate receptors (GluAs). Moreover, the fenestration of the calyx of Held, a hallmark of structural maturation, occurred normally. We thus show that the maintenance of a specialized high frequency synapse in the auditory brainstem occurs in a hardwired, probably genetically encoded, manner with little dependence on synaptic activity.

## Introduction

Ever since the pioneering work of Hubel and Wiesel in the visual system, neuronal activity has been recognized as a key determinant for proper development and maturation of neuronal circuits (Hubel and Wiesel, 1964, 1965; Katz and Shatz, 1996; Blankenship and Feller, 2010; Kirkby et al., 2013; Wang and Bergles, 2015). Recent findings in circuits of the forebrain and neuronal cultures, however, questioned this long-standing theory. The absence of Munc13 or Munc18, both crucial for neurotransmitter release, or cleavage of the SNARE protein VAMP2 (a.k.a. synaptobrevin2) by the expression of TeNT caused only a moderate reduction in synapse numbers (Verhage et al., 2000; Varoqueaux et al., 2002; Sando et al., 2017; Sigler et al., 2017; Quinn et al., 2019). Thus, synapse assembly and maintenance appear to be only partially dependent on neuronal activity but rather rely on cell intrinsic programs. These intriguing findings in forebrain neurons, which are active at moderate frequencies, raise the question whether assembly and maintenance of specialized high frequency synapses is also controlled by cell intrinsic genetic programs rather than by synaptic activity.

A prime high frequency brain area is the auditory brainstem, as the processing of sound features relies on ultrafast and ultraprecise timing of synaptic signals (Grothe et al., 2010). Moreover, spontaneous synaptic activity in the auditory system has been described long before the onset of hearing, suggesting a role in auditory circuit development (Wang and Bergles, 2015; Babola et al., 2018). High frequency, high-fidelity synaptic transmission is achieved by various synaptic specializations, including giant synapses such as the calyx of Held. This glutamatergic axo-somatic synapse arises from globular bushy cells (GBCs) in the ventral cochlear nucleus (VCN) and terminates on principal neurons of the contralateral medial nucleus of the trapezoid body (MNTB) (Borst and Soria van Hoeve, 2012). The calyx undergoes profound morphological and functional maturations, as it develops from a bouton-type synapse [postnatal day 2 (P2)] to its cup-shaped juvenile form (P6) and later, after the onset of hearing, into the fenestrated, mature synapse (P14) (Kandler and Friauf, 1993; Hoffpauir et al., 2006; Ford et al., 2009; Borst and Soria van Hoeve, 2012; Holcomb et al., 2013). The role of synaptic activity for the development and maturation of the calyx of Held as well as MNTB principal neurons has so far been investigated in congenitally deaf or cochlea ablated mice and gerbils. Altered synaptic activity due to a lack of neurotransmitter release from inner hair cells of the cochlea during circuit development resulted in the loss of tonotopic organization in the MNTB (Leao et al., 2006; Ford et al., 2009), action potential broadening, increased NMDA receptor expression (Erazo-Fischer et al., 2007) and considerable cell death of MNTB neurons (Hirtz et al., 2011; Satheesh et al., 2012). Moreover, conductive hearing loss caused changes in calyceal morphology as well as in pre- and postsynaptic function (Grande et al., 2014).

However, in deaf or deafened animal models the auditory system is affected as a whole with auditory brainstem neurons generating abnormal spontaneous activity by themselves (Youssoufian et al., 2008). In order to overcome this limitation, we selectively expressed TeNT in the bushy cells of the VCN using Math5-Cre mice, which express Cre-recombinase specifically in these cells. This strategy blocks neurotransmitter release in the calyx of Held without disrupting the complete auditory brainstem circuitry. Electrophysiological analysis revealed a lack of neurotransmitter release from the calyx of Held after 14 days of TeNT expression. Yet, even after another 7 days of synaptic silence, the calyx was not retracted, degraded or altered with respect to AZ number or postsynaptic AMPA receptor composition. We rather observed minor changes in synapse morphology. These findings suggest that the maintenance of the calyx of Held giant synapse is largely independent of synaptic activity but may rather be controlled by cell intrinsic, genetic means.

## Materials and methods

### Animals

All experiments were conducted in accordance with the German federal law and the EU directive 2010/63 for the care and use of laboratory animals. Protocols were approved by the local authorities (Regierungspräsidium Karlsruhe). All experiments were conducted in heterozygous Math5-Cre mice in which the coding sequence of the transcription factor Math5 (a.k.a. Atoh7) is replaced by Cre-recombinase on one allele (Yang et al., 2003). Heterozygous mice were chosen for the experiments since the lack of both Math5 alleles results in latency shifts in auditory brain stem responses and thus an impairment of auditory processing. However, these shifts have not been reported for mice lacking only one Math5 allele (Saul et al., 2008). Mice were stereotactically injected at P6/7 with AAVs encoding either TeNT fused to EGFP or EGFP alone in a Cre-dependent manner. Injected mice were returned to their mothers and kept there until further use. Mice were sacrificed at P20/21 (electrophysiology) or P27/28 (immunohistochemistry, electron microscopy).

### AAV constructs

**FIGURE 1:**
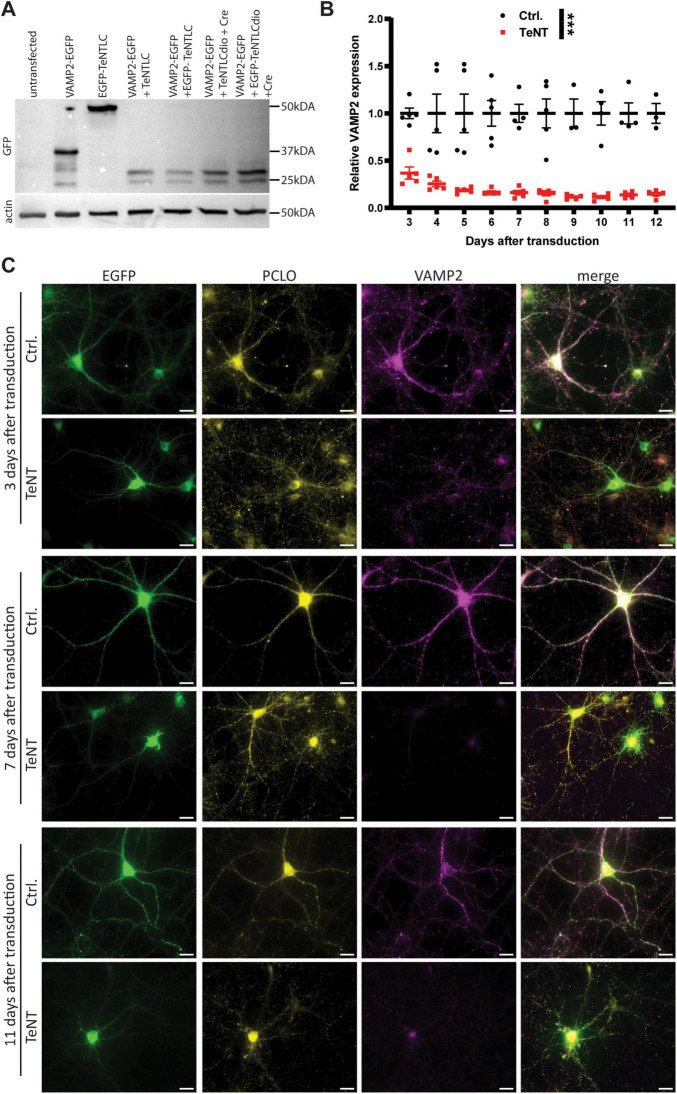
EGFP-TeNT is functional in HEK293 cells and cultured cortical neurons. **(A)** Western Blot analysis of untransfected HEK293 cells (lane 1) and HEK293 cells expressing VAMP2-EGFP (lane 2), EGFP-TeNT (lane 3), VAMP2-EGFP and untagged TeNT (lane 4), VAMP2-EGFP and EGFP-TeNT (lane 5), VAMP2-EGFP, untagged, Cre-dependent TeNT and Cre-recombinase (lane 6) and VAMP2-EGFP, Cre-dependent EGFP-TeNT and Cre-recombinase (lane 7), probed against EGFP. Co-expression of VAMP2-EGFP with any of the TeNT constructs results in a band shift of 8 kDa, which corresponds to the molecular weight of VAMP2 amino acids 1 to 76 which are cleaved of by TeNT. Of note, EGFP tagged versions of TeNT are barely detectable, as soon as VAMP2-EGFP is co-expressed. This is due to a reduction in the amount of TeNT present in the samples. **(B)** Quantification of VAMP2 fluorescence signal in cultured cortical neurons expressing either EGFP only or EGFP-TeNT, normalized to control (5 independent neuronal cultures, for each culture: average of 5 neurons per condition, *p* < 0.001, two-way ANOVA, *p* < 0.05 for each time point, Sidak’s post-hoc test). **(C)** Representative images of cultured cortical neurons expressing either Cre-dependent EGFP or Cre-dependent EFGP-TeNT and Cre-recombinase stained for VAMP2 and Piccolo at 3, 7 and 11 days after transduction. Scale bars are 20 μm. *** = *p* < 0.001.

The coding sequence of tetanus toxin light chain was N-terminally fused to EGFP as described before (Nakashiba et al., 2008), to allow identification of synapses expressing the construct. The fusion protein was cloned between two loxP-sites to render its expression Cre-dependent. EGFP alone, cloned between two loxP-sites served as a control. Cre-dependent expression of both constructs was under the control of the CAG or the hsyn promotor. The constructs were packed in chimeric AAV particles of the serotypes 1 and 2, as described previously (Körber et al., 2015). The functionality of EGFP-TeNT was assessed by Western Blot and immunocytochemistry (Figure 1). Co-expression of the EGFP-TeNT construct and EGFP-tagged VAMP2 in HEK293 cells resulted in an apparently complete cleavage of VAMP2, judged by the shift of the VAMP2 signal from 40 kDa (VAMP2: 13 kDa, EGFP: 27 kDa) to 32 kDa (Figure 1A, VAMP2 amino acids 1–76: ∼8 kDa). To ensure that functionality of the tagged, Cre-dependent TeNT was not limited to HEK293 cells, we examined the effect of TeNT-expression on VAMP2 levels in cultured cortical neurons by immunocytochemistry, using an anti-VAMP2 antibody that does not recognize the cleaved form (Figures 1B, C; Sando et al., 2017). We observed a marked reduction in VAMP2 signal in TeNT-expressing neurons already 3 days after transduction (Figure 1C). VAMP2 levels plateaued 6 days after transduction with no further reduction for the next 6 days (Figure 1B). The remaining VAMP2 signal originated mostly from structures in the somata of the neurons, e.g., the ER, in which VAMP2 is shielded from TeNT (Figure 1C). In accordance with previous reports (Hoogstraaten et al., 2020), TeNT-expressing synapses, identified by immunostaining against Piccolo, were apparently devoid of VAMP2 signal by P6 (Figure 1C).

### Western blotting

HEK293 cells were transfected with plasmids encoding EGFP-tagged VAMP2 and various versions of TeNT (tagged or untagged, Cre-dependent or Cre-independent) using the polyethylenimine method. Three days after transfection, cells were lysed in RIPA buffer supplemented with protease inhibitor. Membranes were pelleted and the supernatant was subjected to SDS-PAGE and semi-dry Western Blotting. Proteins were probed with a primary antibody against EGFP (rabbit polyclonal, target species: species independent, dilution 1:2000, Cat. no.: PABG1; Proteintech, Martinsried, Germany). β-actin served as a loading control (mouse monoclonal, target species: mouse, rat, zebrafish, human, dilution 1:5000, Cat. no.: 251011, Synaptic Systems). Appropriate secondary antibodies conjugated to HRP (Biorad or Proteintech) were used at dilutions of 1:3000 (EGFP) or 1:15000 (β-actin). Protein signal was visualized using SuperSignal West Pico Plus Chemoluminescent Substrate (Cat. no. 34577, ThermoFischer) and an iBright Imaging System (ThermoFischer).

### Immunocytochemistry

Cultured cortical neurons transduced with AAVs encoding either membrane-bound EGFP or Cre-dependent EGFP-TeNT and Cre-recombinase (separate AAVs) were fixed (4% PFA in PBS) for 5 min, 3 to 12 days after transduction. Cells were washed three times in PBS and stored at 4°C until further use. Blocking and permeabilization were performed in blocking buffer (1% normal goat serum, 0,2% Triton-X-100 in PBS) for 90 min. Incubation with primary antibodies against GFP (chicken polyclonal; dilution 1:1000; target species: species independent, Cat. no. ab13970, Abcam), Piccolo (guinea pig polyclonal, dilution 1:1000; target species: rat, mouse; Cat. no.: 142104; Synaptic Systems) and VAMP2/Synaptobrevin2 (mouse monoclonal; dilution 1:1000; target species: human, rat, mouse, hamster; Cat. no. 104211, Synaptic Systems) was performed in blocking buffer for 2 h. After washing with PBS (three times, 10 min), appropriate secondary antibodies coupled to Alexa-dyes (dilution 1:1000; Thermo Fischer) were applied for 45 min in blocking buffer. Thereafter, cells were washed 3 times in PBS and mounted in Mowiol. All incubation steps were performed at room temperature under gentle agitation. Images were acquired using a widefield fluorescence microscope (DM6000, Leica) equipped with a 63× HCX PL APO (1.45 NA) objective. Relative VAMP2 fluorescence signal was analyzed with ImageJ.

### Stereotaxic injections

AAV particles were stereotactically injected into the VCN of anesthetized Math5-Cre mice of either sex at P6/7 as described earlier (Vasileva et al., 2012). In brief, mice were deeply anesthetized using isoflurane and aligned in the stereotactic apparatus (Kopf Instruments, Tujunga, CA, USA). A craniectomy was performed and ∼2 μl of virus solution were evenly distributed to the following coordinates relative to bregma and midline (*x*, *y*, in mm): 0.5, –4.5; 0.55, –4.5; 0.6, –4.5; 0.45, –5.1; 0.45, –5.2 using a custom build manipulator (Wimmer et al., 2004). Z- and A-positions were kept constant at 0.45 mm and 6.5 mm, respectively. After the surgery, mice recovered within minutes and were returned to their mothers.

### Preparation of fixed brain slices

Mice were deeply anesthetized with Pentobarbital (500 mg/kg bodyweight) at P27/28 and transcardially perfused with 15 ml of PBS followed by 15 ml of Zamboni’s solution (2% PFA, 15% picric acid in 0.15 M phosphate buffer). Brains were removed from the skull, post-fixed at 4°C for 2–4 h (immunohistochemistry) or over night (electron microscopy) and stored in 30% sucrose/PBS at −20°C until further usage.

### Immunohistochemistry

Coronal sections of 60–80 μm thickness were cut on a vibratome (VT1000S, Leica) and antibody staining was performed as described previously (Ebbers et al., 2015). Briefly, slices were washed three times in PBS before incubation in blocking solution (3% bovine serum albumin, 10% goat serum and 0.3% Triton in PBS) for 1 h at room temperature. The following primary antibodies were applied in carrier solution (1% bovine serum albumin, 1% normal goat serum, and 0.3% Triton X-100 in PBS) and incubated over night at 4°C: anti-Piccolo (guinea pig polyclonal, dilution 1:200; target species: rat, mouse; Cat. no.: 142104; Synaptic Systems), anti-GFP (chicken polyclonal; dilution 1:1000; target species: species independent; Cat. no.: ab13970, Abcam), anti-NeuN (mouse monoclonal, dilution 1:250, target species: avian, chicken, ferret, human, mouse, pig, rat, salamander, Cat. no: mab377, Sigma-Aldrich), anti-vGlut1 (guinea pig polyclonal, dilution 1:1000, target species: rat, Cat. no.: ab5905, Sigma-Aldrich), anti-GluA1 (mouse monoclonal; dilution 1:500; target species: rat, mouse, human; Cat. no.: 182011; Synaptic Systems) and anti-GluA4 (rabbit polyclonal; dilution 1:500; target species: rat, mouse, chicken, human, chimpanzee; Cat. no.: AB1508; Merck). Subsequently, slices were washed in PBS and incubated with appropriate Alexa-coupled secondary antibodies in carrier solution (dilution 1:1000; 1.5 h; room temperature). Slices were again rinsed with PBS and mounted.

Stained brainstem sections were examined by confocal microscopy using a Leica TCS SP8 microscope equipped with a 63× HCX PL APO (1.45 NA) objective. Overview images were acquired on an Olympus BX63 microscope with a 20× UPlanSApo (0.75 NA) objective. Fluorescence signals of EGFP and immunolabeled structures were automatically detected and segmented using trained classifiers in ilastik software (Berg et al., 2019). Quantitative structural 3D analysis, cell counting and colocalization analysis were performed using arivis Vision 4D software (Zeiss).

### Preparation of acute brainstem slices

Mice were rapidly decapitated at P20/21. Brains were removed in ice-cold slicing solution containing (in mM): 125 NaCl, 25 NaHCO_3_, 2.5 KCl, 1.25 NaH_2_PO_4_, 3 myoinositol, 2 Na-pyruvate, 0.4 ascorbic acid, 0.1 CaCl_2_, 3 MgCl_2_ and 25 glucose aerated with carbogen (5% CO_2_ in O_2_). Brainstem slices of 200 μm thickness were prepared on a vibratome (VT1200S, Leica) and stored in artificial cerebrospinal fluid (ACSF) (in mM: 125 NaCl, 25 NaHCO_3_, 2.5 KCl, 1.25 NaH_2_PO_4_, 2 CaCl_2_, 1 MgCl_2_ and 25 glucose aerated with carbogen, pH 7.3) at 37°C for 45 min and at room temperature (22 ± 1°C) thereafter.

### Electrophysiology

Whole-cell patch-clamp recordings were established from MNTB principal neurons contacted by fluorescently labeled calyces, using an EPC-10/2 amplifier controlled by PatchMaster software (HEKA, Reutlingen, Germany). Recordings were performed in ACSF supplemented with 2 μM strychnine (Sigma Aldrich) using pipettes pulled from thick-walled borosilicate glass (Cat. no.: 1807515, Hilgenberg, Malsfeld, Germany) which had open tip resistances of 2–3 MΩ. The pipette solution contained (in mM): 130 Cs gluconate, 10 CsCl, 10 HEPES, 10 TEA-Cl, 5 Na_2_-phosphocreatine, 5 EGTA, 4 Mg-ATP and 0.3 GTP (pH 7.2). The holding potential was −70 mV. Series resistances ranged from 3 to 6 MΩ and were compensated for by > 90%. Currents were digitized at 100 kHz and Bessel-filtered at 2.9 kHz. All experiments were performed at room temperature. Synaptic responses were elicited by bipolar pulses (100 μs, 50 V) applied via a bipolar stimulation pipette (Θ-barrel, open tip diameter ∼3 μm) filled with ACSF that was positioned in the immediate vicinity of the labeled calyx hemi-node. Data were analyzed using custom-written IGOR (Wavemetrics, Lake Oswego, OR, USA) routines.

### Correlated light and electron microscopy

Fixed brain slices were prepared as described above. MNTBs containing fluorescently labeled calyces were excised from coronal brainstem sections and stained with DAPI. Confocal image stacks were obtained from both sides of the tissue blocks using a Leica TCS SP8 microscope equipped with a 20× HC PL APO CS2 (0.75 NA) objective and denoised.

Afterward, the tissue blocks were prepared for scanning electron microscopy as described previously (Horstmann et al., 2012). In brief, tissue blocks were incubated in cacodylic acid (100 mM) for 30 min and post-fixed in 1.5% potassium ferricyanide and 2% osmium tetroxide (1 h, on ice). Samples were washed with water, dehydrated in an ascending series of alcohol, incubated in propylene oxide/epoxy (1:1) over night and embedded in epoxy resin (polymerization: 36 h, 60°C). Ribbons of ultrathin sections (40 nm, ∼150 sections/ribbon) were cut through the MNTB on an Ultracut S microtome (Leica) equipped with a diamond knife angled at 45° (Diatome, Biel, Switzerland) and collected on clean, glow discharged silicon wafers (SiMat Silicon Materials, Landsberg, Germany). The sections were exposed to chloroform vapor to neutralize tissue compression due to sectioning, dried and stained using a modified Reynolds-procedure [saturated uranyl acetate (16 min) followed by lead citrate (8 min)]. Scanning electron microscopy was performed using a LEO Gemini 1530 equipped with a field emission gun and an ATLAS scanning generator (Zeiss). Calyces that showed fluorescent labeling in confocal image stacks were identified in EM sections based on their position relative to characteristic groups of nuclei. Electron micrographs of identified synapses were taken at a pixel size of 3.8 nm using the InLens detector at the following settings: 3.6 mm working distance, 30 μm aperture and 2 keV acceleration voltage.

Electron microscopy experiments and analysis were performed in a double-blind manner. AZs and synaptic vesicles (SVs) were manually traced using OpenCAR software (Sätzler et al., 2002). AZs were identified by the presence of an opposing postsynaptic density and of SVs in the vicinity, thereby avoiding confusion with puncta adherentia (Sätzler et al., 2002). The perpendicular distance between individual SVs and the AZ was calculated using OpenCARe (Sätzler et al., 2002).

### Statistical analysis

Statistical significance was assigned by unpaired, two-tailed Student’s *t*-test or two-way ANOVA followed by Sidak’s post-hoc test using Prism 10 software (Graphpad software, La Jolla, CA). Significant differences are marked by asterisks (* = *p* < 0.05, ** = *p* < 0.01, *** = *p* < 0.001). Data are presented as mean ± SEM.

## Results

**FIGURE 2:**
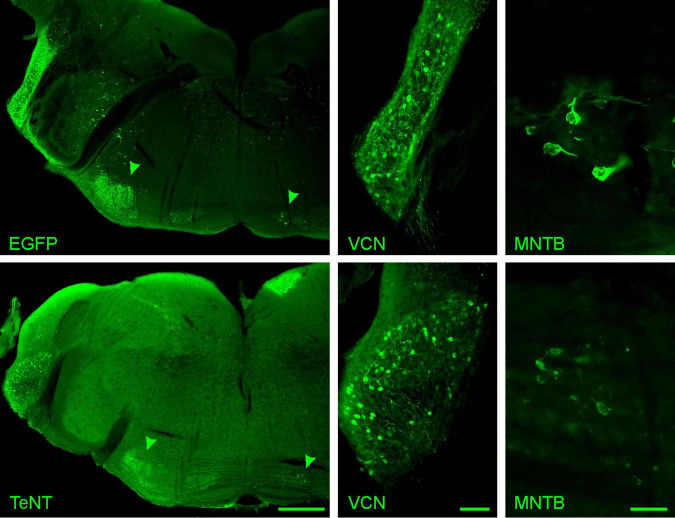
Tetanus toxin is expressed in bushy cells of the VCN. Cre-dependent expression of EGFP-tagged TeNT and EGFP control in the auditory brainstem of a Math5-Cre mouse are visualized at P28 by EGFP fluorescence. Top row: EGFP control, bottom row: EGFP-tagged TeNT. Left: overview of the auditory brainstem including the VCN and the superior olivary complex. Green arrowheads indicate the projection areas of spherical and globular bushy cells, the ipsilateral LSO and the contralateral MNTB, respectively. Middle: Close-up of the VCN showing fluorescent, tagged TeNT- or EGFP-expressing cells. The pattern of fluorescently labeled cells is consistent with Math5-expressing bushy cells (Saul et al., 2008). Right: Close-up of the contralateral MNTB showing calyces expressing either tagged TeNT or EGFP only. Scale bars are 500 μm (left) and 100 μm (middle, right).

**FIGURE 3:**
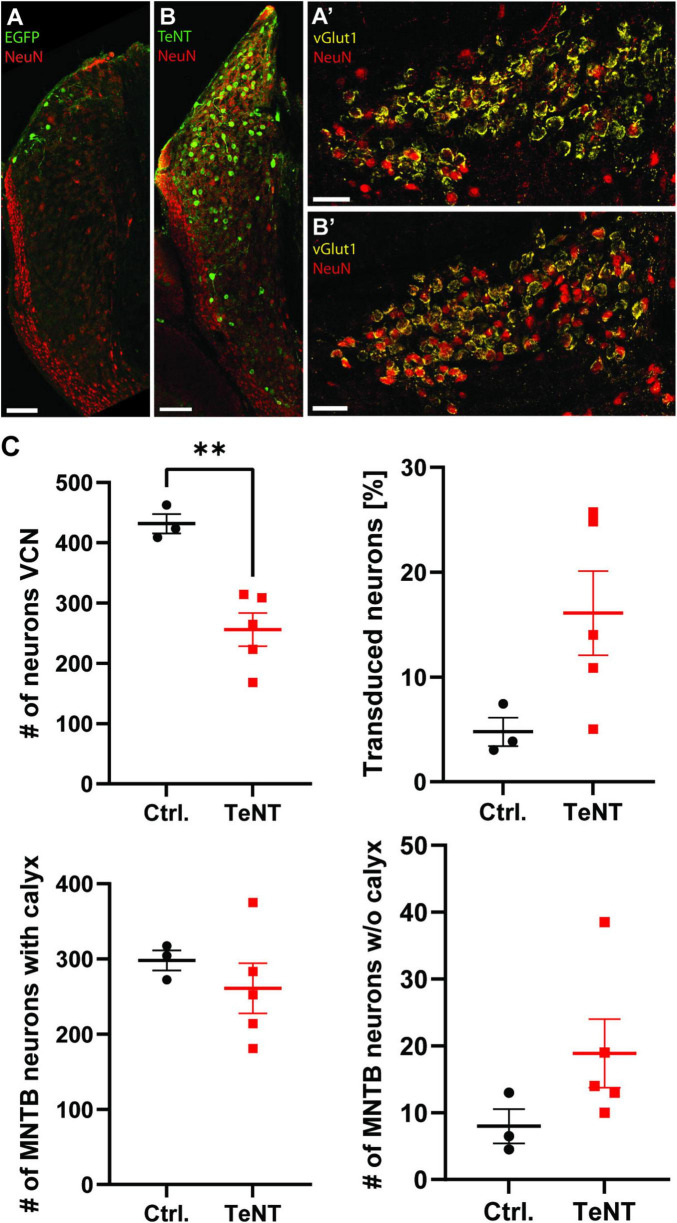
Tetanus toxin expression leads to a reduction in bushy cell number in the VCN without affecting calyx numbers in the VCN. **(A,B)** Representative images of the VCN of Math5-Cre mice expressing either EGFP or TeNT stained for the pan-neuronal marker NeuN. **(A’,B’)** Representative images of the MNTB of Math5-Cre mice expressing either EGFP **(A’)** or TeNT **(B’)** stained for the pan-neuronal marker NeuN and the calyceal marker vGlut1. Images represent maximal projections of confocal stacks spanning 20 μm in depth. **(C)** Quantification of the number of neurons in the VCN, the transduction efficiency of both AAV constructs, the number of calyces in the contralateral MNTB and the number of MNTB neurons not contacted by a vGlut1-labeled calyx. (*n* = 2–5 slices per animal, 3–5 mice, student’s *t*-test). **p < 0.01.

To investigate how the absence of synaptic activity affects the calyx of Held, we used TeNT to abolish synaptic transmission selectively at this synapse. TeNT cleaves the SNARE proteins synaptobrevin 1 and synaptobrevin 2 (a.k.a. VAMP1 and VAMP2, respectively), thereby preventing the formation of the SNARE complex and thus synaptic vesicle (SV) fusion (Link et al., 1992; Humeau et al., 2000). Specific silencing of the calyx was achieved by Cre-dependent TeNT expression in the bushy cells of the VCN of Math5-Cre mice (Yang et al., 2003; Saul et al., 2008). Therefore, AAV particles coding either for EGFP-tagged TeNT or EGFP only were stereotactically injected into the VCN at P6/7. Effectivity of functional silencing of the calyx was assessed at P20/21, after 14 days of TeNT expression, while structural changes were examined at P27/28, 21 days after AAV injection and at least 7 days after the cessation of synaptic transmission. At P28, numerous cells expressing EGFP or TeNT were detected throughout the VCN, in accordance with the distribution of bushy cells (Figure 2; Saul et al., 2008). Moreover, projections of fluorescently labeled TeNT-expressing VCN cells were consistent with those of Math5-expressing spherical bushy cells [ipsilateral lateral superior olive (LSO)] and globular bushy cells (contralateral MNTB) (Figure 2, arrow heads; Saul et al., 2008). We next examined, if transduction of either AAV was more effective and if expression of TeNT led to neuronal cell death. Therefore, we stained neurons in the VCN with the pan-neuronal marker NeuN and determined the total number of neurons in the VCN and the number of neurons expressing either EGFP alone or TeNT (Figures 3A, B). VCNs of mice expressing TeNT contained fewer neurons compared to mice expressing only EGFP (Figure 3C, control: 432 ± 16 cells/VCN, average of 3–4 slices per mouse, 3 mice; TeNT: 256 ± 27 cells/VCN, average of 2–4 slices per mouse, 5 mice, *p* = 0.0037, student’s *t*-test). Interestingly, we found higher transduction rates in mice injected with TeNT-encoding AAVs (Figures 3A–C, control: 5% ± 1%, average of 3–4 slices per mouse, 3 mice; TeNT: 16% ± 4%, average of 2–4 slices per mouse, 5 mice, *p* = 0.083, student’s *t*-test). Next, we examined if the neuron loss in the VCN also results in a loss of calyces in the contralateral MNTB. Therefore, we labeled calyces with the presynaptic marker vGlut1 and determined the number of calyces per MNTB and the number of NeuN-labeled cells that were not contacted by a calyx synapse (Figures 3A’, B’, C). We did not find a reduction in the number of labeled calyces in the MNTB of mice injected with TeNT, as compared to control (control: 298 ± 13 calyces/MNTB, average of 2 slices per mouse, 3 mice; TeNT: 261 ± 33 calyces/MNTB, average of 2 slices per mouse, 5 mice, *p* = 0.45, student’s *t*-test). However, when we counted the number of MNTB neurons that were not contacted by a large, calyx-like vGlut1-expressing synapse, we observed more such neurons in MNTBs of TeNT-expressing mice, although this trend did not reach significance (Figures 3A’, B’, C; control: 8 ± 3 cells/MNTB, average of 2 slices per mouse, 3 mice; TeNT: 19 ± 5 calyces/MNTB, average of 2 slices per mouse, 5 mice, *p* = 0.17, student’s *t*-test). Of note, we observed multiple calyces that were contacting MNTB principal neurons not labeled by NeuN. We thus conclude that TeNT expression in the VCN results in cell loss in the VCN of TeNT expressing mice, but not in a loss of calyces under our experimental conditions. TeNT thus provides a suitable tool to assess the effects of synaptic silence on the structure of the calyx.

### TeNT practically abolishes synaptic transmission at the calyx of Held

**FIGURE 4:**
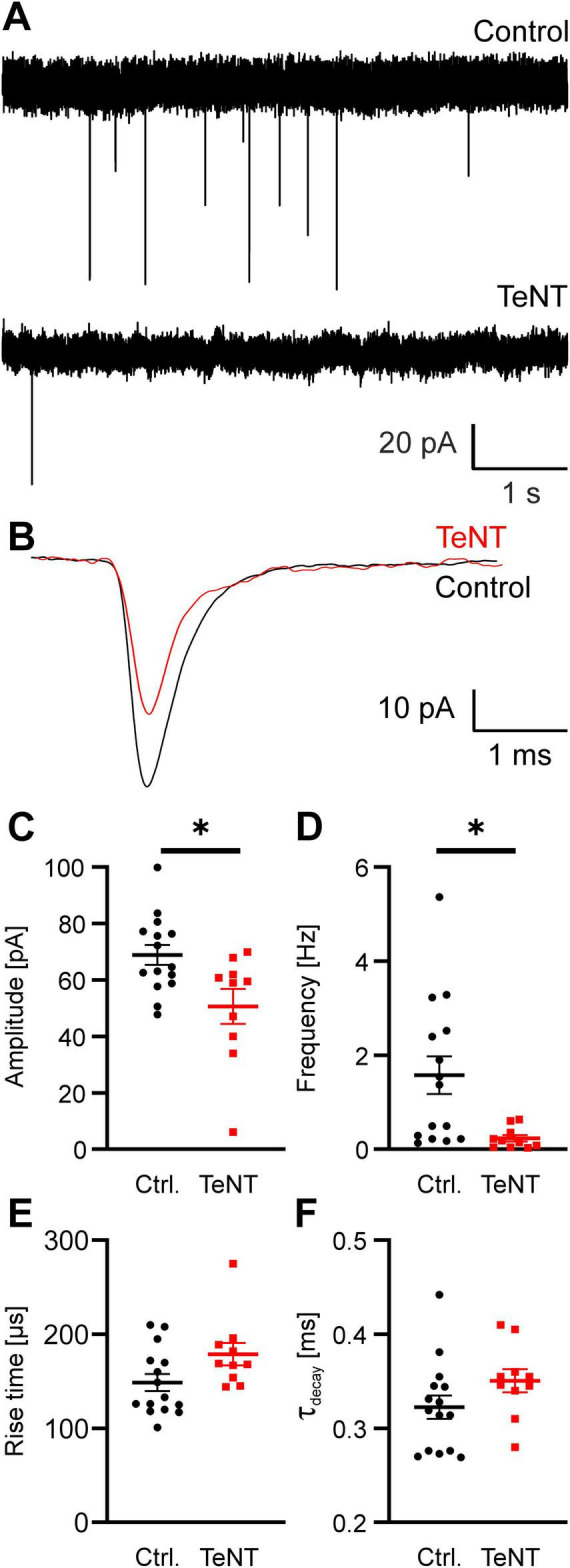
Expression of TeNT impairs spontaneous neurotransmitter release at the calyx of Held. **(A)** Representative sample current traces obtained from MNTB neurons at P21 show a drastic reduction in sEPSC frequency. **(B)** Overlay of averaged sEPSCs obtained from the same cells as in **(A)**. **(C–F)** Quantification of sEPSC properties (amplitude, frequency, rise time and decay time constant, *n* = 10–15, student’s *t*-test). * = *p* < 0.05.

Synaptic silencing by TeNT was assessed by recording spontaneous and evoked EPSCs (sEPSCs and eEPSCs, respectively). First, we recorded sEPSCs from MNTB principal neurons contacted by fluorescently labeled TeNT or EGFP-control calyces at P20/21. sEPSC frequency was greatly diminished in MNTB neurons contacted by TeNT-expressing calyces as compared to controls (control: 1.57 ± 0.40 Hz, *n* = 15 calyces from 7 mice, TeNT: 0.23 ± 0.07 Hz, *n* = 10 calyces from 7 mice, *p* = 0.013, student’s *t*-test) (Figures 4A, D). Nevertheless, we still observed some residual sEPSCs in MNTB neurons contacted by TeNT calyces. These sEPSCs were significantly smaller and had a tendency toward slower kinetics than those obtained from MNTB cells contacted by control calyces (amplitude: control: 69 ± 4 pA, *n* = 15 calyces from 7 mice, TeNT: 51 ± 6 pA, *n* = 10 calyces from 7 mice, *p* = 0.012; risetime: control: 149 ± 9 μs, *n* = 15 calyces from 7 mice, TeNT: 179 ± 12 μs, *n* = 10 calyces from 7 mice, *p* = 0.05; decay time constant: control: 0.323 ± 0.012 ms, *n* = 15 calyces from 7 mice, TeNT: 0.351 ± 0.012 ms, *n* = 10 calyces from 7 mice, *p* = 0.14, student’s *t*-tests) (Figure 4). This would be consistent with a non-calyceal origin as described previously (Hamann et al., 2003). However, this previous study has not examined the properties of non-calyceal sEPSCs explicitly. Recordings of evoked non-calyceal EPSCs suggest that the rise time of these inputs is ∼3 times slower than that of calyceal EPSCs (rise time extra-calyceal EPSCs: 616 ± 85 μs; Hamann et al., 2003). Since the difference in sEPSC rise time observed here is only a trend and not as pronounced as that previously described for non-calyceal inputs, we cannot exclude that some spontaneous release from the calyx remains in TeNT expressing calyces (see discussion).

**FIGURE 5:**
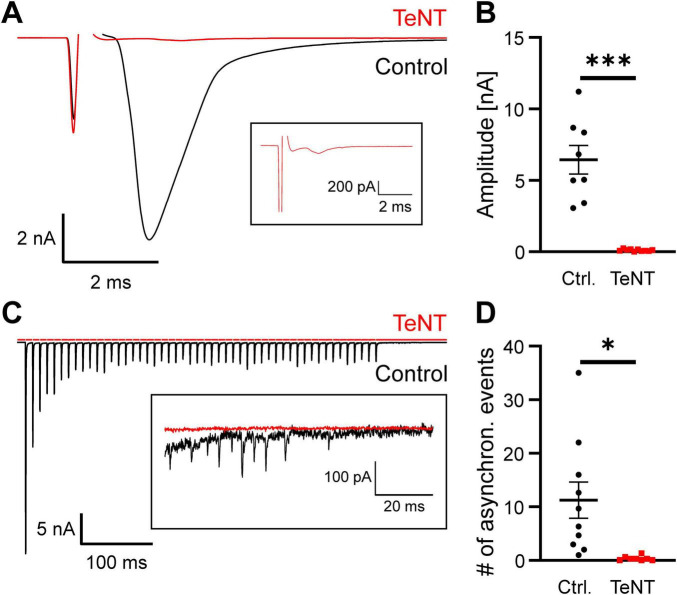
Expression of TeNT abolishes evoked neurotransmitter release at the calyx of Held. **(A)** Representative sample current traces obtained from MNTB neurons at P21 demonstrating the absence of calyceal EPSC in response to direct electrical stimulation of a TeNT-expressing axon. Inset: Detail of the recording from the TeNT-expressing calyx. **(B)** Quantification of evoked EPSC amplitude (*n* = 8–10, student’s *t*-test). **(C)** Representative sample current traces obtained from MNTB neurons at P20/21 in response to trains of 50 electrical stimuli at 100 Hz delivered directly to TeNT- or EGFP-expressing axons. Inset: Detail of the 100 ms after the cessation of the stimulus trains of the recordings shown at higher scale. **(D)** Quantification of the number of miniature-like, asynchronous events in the 100 ms after the cessation of a 100 Hz stimulus train (*n* = 8–10, student’s *t*-test). * = *p* < 0.05, *** = *p* < 0.001.

Based on the sEPSC recordings, we cannot formally rule out that some neurotransmitter release occurs from the calyx in the presence of TeNT. To demonstrate synaptic silencing of the calyx of Held, we performed a second set of experiments and examined evoked neurotransmitter release in response to direct electrical stimulation. At P20/21, typical calyceal EPSCs in the nA-range with fast kinetics (Borst and Soria van Hoeve, 2012) were recorded from 8 out of 8 control calyces, but none of the 10 TeNT-expressing ones (EPSC amplitude: control: 6.45 ± 1.0 nA, *n* = 8 calyces from 7 mice, TeNT: 0.11 ± 0.02 nA, *n* = 10 calyces from 9 mice, *p* < 0.0001, student’s *t*-test) (Figure 5). Occasionally observed small inward currents had waveforms that did not resemble EPSCs and may be artifacts of the close-by stimulation (Figure 5A, inset). Although TeNT-expressing calyces did not show typical EPSCs in response to a single electric stimulus, high frequency stimulation, which resembles the physiological activity pattern during sound perception (Kopp-Scheinpflug et al., 2008), could result in a build-up of presynaptic calcium which in turn could lead to fusion of more release-reluctant SVs. We therefore applied 100 Hz stimulus trains (50 stimuli) to calyces expressing either TeNT or EGFP and recorded the postsynaptic EPSCs at P20/21. Train-elicited EPSC from MNTB principal cells contacted by control calyces showed typical short-term depression (STD) with steady state EPSCs that were still in the nA-range (Figure 5C, *n* = 10 calyces from 6 mice). Application of 100 Hz stimulus trains to TeNT-expressing calyces did not result in detectable EPSCs (Figure 5C, *n* = 8 calyces from 4 mice). Finally, we investigated whether TeNT expression also abolished asynchronous release. Therefore, we determined the number of miniature-like EPSCs in the 100 ms after the ending of the 100 Hz stimulus trains. Control synapses showed variable degrees of asynchronous release while it was virtually absent from TeNT-expressing calyces (Figure 5C inset and Figure 5D, # of miniature-like events: control: 11.2 ± 3.4 events, *n* = 10 calyces from 6 mice, TeNT: 0.4 ± 0.2 events, *n* = 8 calyces from 4 mice, *p* = 0.012, student’s *t*-test). MNTB principal neurons contacted by TeNT-expressing synapses occasionally showed a single miniature-like event during these 100 ms. However, this could also represent spontaneous release from non-calyceal inputs to the MNTB principal cell (see discussion). The lack of evoked as well as asynchronous neurotransmitter release from the calyx thus confirms its silence and thereby the effectiveness of TeNT after 14 days of expression.

### Prolonged synaptic silence induces only minor changes in synapse morphology

**FIGURE 6:**
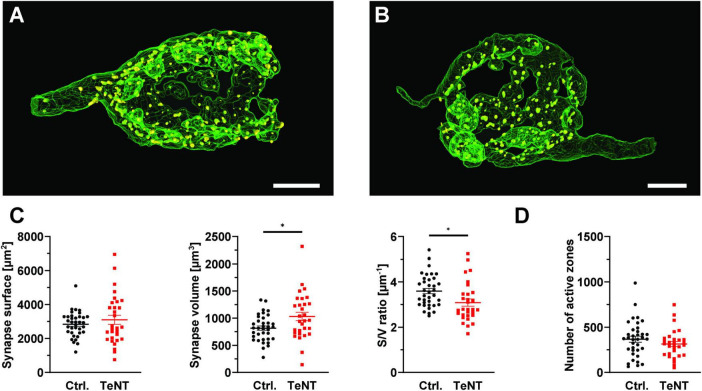
Prolonged expression of TeNT in the calyx of Held has minor impact on synapse morphology. **(A,B)** Representative 3D reconstructions of control **(A)** and TeNT-expressing **(B)** calyces, obtained from confocal image stacks using the fluorescent signal of EGFP-labeled TeNT or cytosolic EGFP (control) at P28. Yellow structures represent AZs identified by immunostainings against Piccolo. **(C)** Quantification of synapse morphology (synapse surface, synapse volume, surface-to-volume ratio, *n* = 30–36, student’s *t*-test). **(D)** Quantification AZ numbers at P27/28 (*n* = 30–36, student’s *t*-test). *p < 0.05; Scale bars in **(A,B)**: 5 μm.

Having established synaptic silence at the calyx of Held after 14 days of TeNT expression, we next examined whether prolonged absence of synaptic transmission causes structural changes or degradation of the calyx. Therefore, we performed confocal imaging of TeNT and control (EGFP only) calyces that were immunolabelled for the AZ marker Piccolo (Gundelfinger et al., 2016) at P27/28, after at least 7 days of synaptic silence. Calyces were 3D reconstructed based on the EGFP signal. We did not observe grossly changed calyces even after 21 days of TeNT expression. However, TeNT calyces showed a slight increase in synaptic volume as compared to controls (control: 816 ± 40 μm^3^, *n* = 36 calyces from 3 mice, TeNT: 1030 ± 78 μm^3^, *n* = 30 calyces from 3 mice; *p* = 0.013, student’s *t*-test) while the surface of the calyx remained constant (control: 2847 ± 122 μm^2^, *n* = 36 calyces from 3 mice, TeNT: 3103 ± 267 μm^2^, *n* = 30 calyces from 3 mice; *p* = 0.36, student’s *t*-test). This resulted in a thickening of the calyx of Held, as indicated by the decrease in surface/volume-ratio (control: 3.6 ± 0.12 μm^–1^, *n* = 36 calyces from 3 mice, TeNT: 3.09 ± 0.16 μm^–1^, *n* = 30 calyces from 3 mice; *p* = 0.01, student’s *t*-test) (Figures 6A–C). These minor changes in synaptic morphology were not accompanied by a change in AZ number as the number of Piccolo-labeled immunoreactive clusters remained constant (control: 368 ± 33 clusters/calyx, *n* = 36 calyces from 3 mice, TeNT: 315 ± 28 clusters/calyx, *n* = 30 calyces from 3 mice; *p* = 0.24, student’s *t*-test) (Figure 6D). Our results thus indicate that the morphology of the calyx of Held as well as the number of AZs are largely independent of synaptic activity.

### The AMPA receptor composition at the calyx of Held synapse is independent of synaptic activity

**FIGURE 7:**
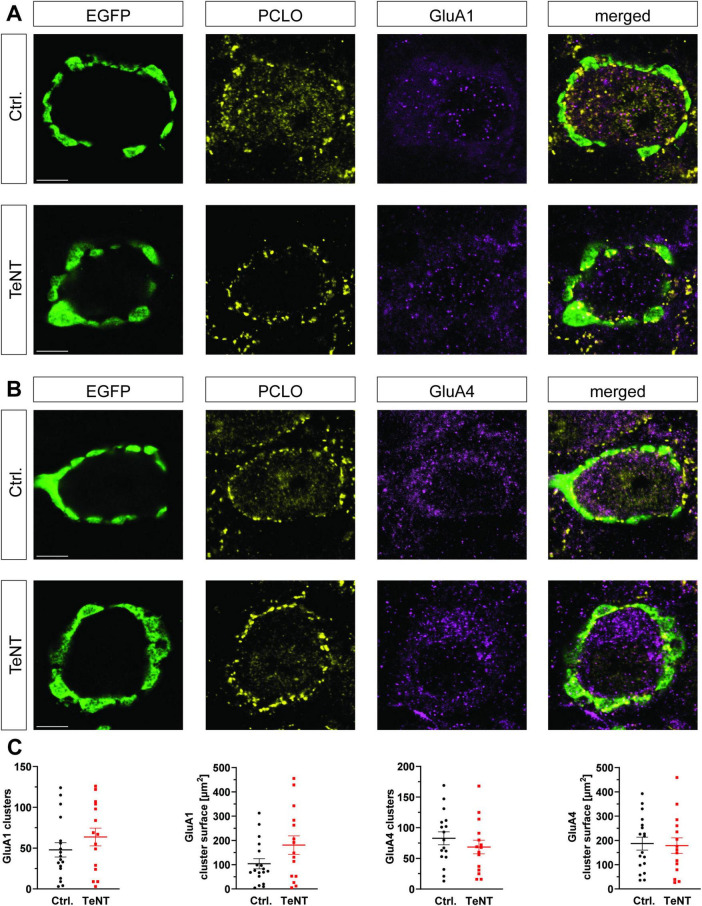
TeNT expression does not change postsynaptic AMPA receptor composition. **(A,B)** Representative confocal images (single plane) of calyces expressing TeNT or EGFP stained for Piccolo and either GluA1 **(A)** or GluA4 **(B)** at P28. The immunoreactive signal inside the MNTB principal neurons represents ongoing protein synthesis/processing. It is not used for quantification. **(C)** Quantification of number and size of synaptically localized GluA clusters (*n* = 15–18, student’s *t*-test). Scale bars: 5 μm.

The lack of major morphological changes at the calyx prompted us to examine if the absence of synaptic activity leads to alterations on the postsynaptic side of the synapse. Therefore, we investigated the composition of the postsynaptic AMPA receptors by staining for the subunits GluA1 and GluA4 in TeNT and control synapses. GluA4 is highly expressed at the mature calyx of Held (Yang et al., 2011), while the juvenile synapses also possess GluA1 receptors (Borst and Soria van Hoeve, 2012). Synaptic localization of GluA clusters was determined by partial overlap of immunoreactive signals for the GluA subunit and Piccolo. We did not observe differences in the number or size of synaptic GluA4 clusters in TeNT and control synapses (number: control: 83 ± 11 clusters/calyx, *n* = 17 calyces from 3 mice, TeNT: 69 ± 11 clusters/calyx, *n* = 15 calyces from 3 mice, *p* = 0.36, student’s *t*-test; size: control: 184 ± 28 μm^2^, *n* = 17 calyces from 3 mice, TeNT: 178 ± 32 μm^2^, *n* = 15 calyces from 3 mice, *p* = 0.89, student’s *t*-test) (Figures 7B, C). Likewise, synaptic AMPA receptor clusters containing the juvenile subunit GluA1 in MNTB neurons contacted by TeNT-expressing calyces remained constant in number and size compared to control synapses. Notably, GluA1 clusters in TeNT synapses tended to be bigger than those in control ones, although this trend did not reach significance (number: control: 48 ± 9 clusters/calyx, *n* = 18 calyces from 3 mice, TeNT: 64 ± 11 clusters/calyx, *n* = 15 calyces from 3 mice, *p* = 0.27, student’s *t*-test; size: control: 104 ± 21 μm^2^, *n* = 18 calyces from 3 mice, TeNT: 181 ± 38 μm^2^, *n* = 15 calyces from 3 mice, *p* = 0.07, student’s *t*-test) (Figures 7A, C). The lack of synaptic transmission thus did not result in a pronounced compensatory incorporation of the juvenile GluA1 subunit into the calyx of Held synapse.

### TeNT expression leads to a loss of SVs close to the AZ without affecting AZ length

Although our results so far suggest that the structure of the calyx is largely independent of synaptic transmission, we next examined the ultrastructure of AZ in calyces expressing TeNT. Therefore, we performed corelative light and electron microscopy (CLEM) of identified TeNT and control calyces.

**FIGURE 8:**
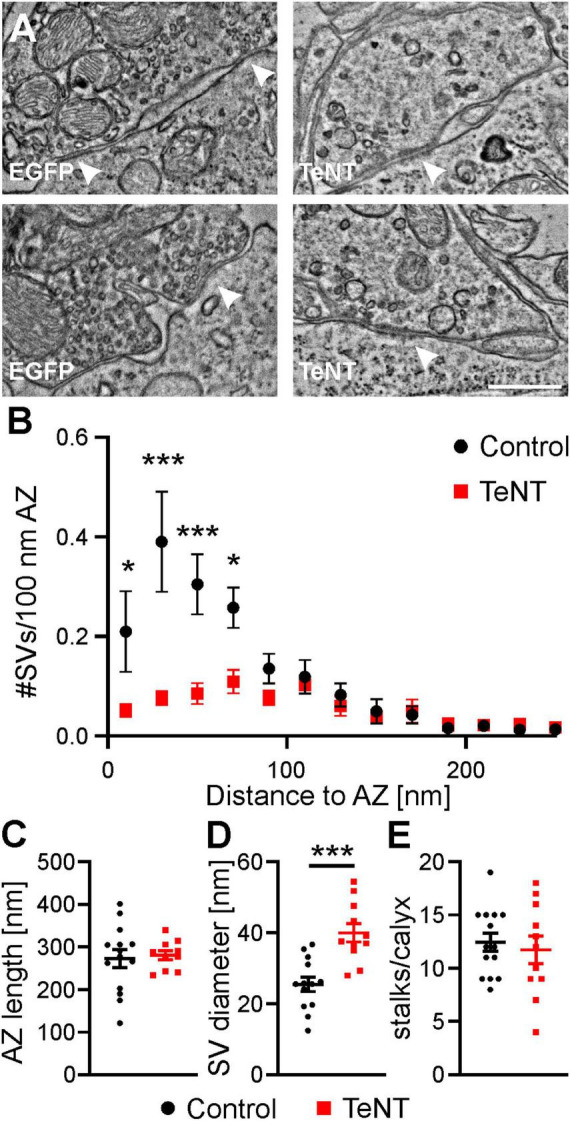
TeNT expression depletes membrane proximal SVs but does not change AZ length. **(A)** Representative electron micrographs of AZs from control and TeNT-expressing calyces, as identified by CLEM. Arrow heads indicate AZs. **(B)** Distribution of SV to AZ distances at AZs from control and TeNT-expressing calyces (*n* = 10–14 calyces, two-way ANOVA followed by Sidak’s post-hoc test). **(C–E)** Quantification of AZ length, SV diameter and the number of stalks per calyx (*n* = 10–14 calyces, student’s *t*-test). Scale bar in **(A)**: 500 nm. * = *p* < 0.05; *** = *p* < 0.001.

In line with results presented above, we did not observe alterations in the gross morphology of TeNT-expressing calyces, as indicated by the number of calyx stalks (control: 12.4 ± 0.8 stalks/calyx, *n* = 14 calyces from 3 mice, TeNT: 11.7 ± 1.3 stalks/calyx, *n* = 10 calyces from 3 mice; *p* = 0.65, student’s *t*-test, Figure 8E). Analysis of the ultrastructure of AZs showed no difference in AZ length after TeNT expression (control: 273 ± 21 nm, *n* = 14 calyces from 3 mice, TeNT: 281 ± 11 nm, *n* = 10 calyces from 3 mice, *p* = 0.78, student’s *t*-test) (Figures 8A, C), but a greatly reduced number of SVs (Figures 8A, B). Consistent with our electrophysiological results, the AZs of TeNT calyces showed a prominent loss of the membrane-proximal SVs (0–20 nm from plasma membrane: control: 0.21 ± 0.08 SVs/100 nm AZ, *n* = 14 calyces from 3 mice, TeNT: 0.05 ± 0.01 SVs/100 nm AZ, *n* = 11 calyces from 3 mice, *p* = 0.026, two way ANOVA followed by Sidak’s post-hoc test) (Figures 8A, B), which constitute the ready-releasable pool of SVs (Imig et al., 2014). Additionally, we found a significant increase in the diameter of SVs in TeNT calyces (control: 25 ± 2 nm, *n* = 14 calyces from 3 mice, TeNT: 40 ± 3 nm, *n* = 10 calyces from 3 mice, *p* = 0.0002, student’s *t*-test) (Figure 8D), which may be caused by a higher fraction of slightly bigger SV-like endocytic compartments (Heuser and Reese, 1973) in the potential SVs analyzed. Another surprising finding of our EM analysis was a general reduction in the number of SVs within a distance of ∼100 nm from the presynaptic plasma membrane in TeNT-expressing calyces (Figure 8B). This could either be caused by defects in local SV recycling or by a reduction in the cells ability to supply new SVs to the synapse and thus an excess in SV degradation (see Discussion).

## Discussion

In the present study, we demonstrate that even prolonged absence of synaptic transmission has only minor effects on the morphology and AMPA receptor composition of the calyx of Held synapse. The silencing of the calyx did not interfere with the maintenance and maturation of the calyx, since fenestration, a hallmark of structural maturation, apparently occurred/proceeded regularly in TeNT synapses. Thus, the structural integrity of this specialized high frequency, high-fidelity synapse is largely independent of synaptic activity.

### TeNT expression efficiently prevents neurotransmitter release without affecting calyx survival

Prolonged expression of TeNT in bushy cells resulted in a decrease in neuronal number in the VCN. However, this loss in neurons was not accompanied by a reduction in the number of calyces in the contralateral MNTB, suggesting that the reduction in the number of VCN neurons could be due to a loss of spherical but not globular bushy cells. Spherical bushy cells (SBCs) also express Math5 and therefore Cre-recombinase in the mouse line used. It could be that, for an unknown reason, SBCs are more sensitive to TeNT expression than GBCs and prolonged TeNT expression therefore leads to a loss of SBCs. The viability of GBCs in turn seems to be unaffected by TeNT expression, as we did not observe a reduction in calyx numbers in the contralateral MNTB, even after 21 days of TeNT expression. This apparent lack of effect of TeNT on GBC survival is in accordance with previous results from neuronal cultures and hippocampal CA1 neurons which are resistant to TeNT expression, too (Santos et al., 2017; Hoogstraaten et al., 2020; Adaikkan et al., 2024). In the MNTB, we did observe a number of NeuN-labeled neurons that were not contacted by vGlut1-labeled calyces. Although the number of these calyx-less MNTB neurons is higher in TeNT expressing mice, the effect did not reach significance and the number is far lower than the 30–40% of cells that have been reported previously (Jackson, 2020). The discrepancy to the previous study could be due to the reference (NeuN vs. MAP2), virus system (AAV vs. helper-dependent adenovirus) or, most likely, the day of virus injection (P6/7 vs. P0). Expression of TeNT from P0 will likely interfere with the expansion of the calyx from a bouton type synapse to the juvenile, cup-shaped terminal (Hoffpauir et al., 2006, Holcomb et al., 2013) while TeNT expression under our experimental conditions only becomes effective at a much later stage. In accordance with this assumption, expression of TeNT from P0 results in a large number of abnormally formed calyces (Jackson, 2020), which we did not observe under our experimental conditions. Of note, we also observed vGlut1-labeled calyceal structures that contacted MNTB neurons devoid of NeuN signal, which is probably due to an insufficient NeuN labeling by the primary antibody and occurs independent of the construct expressed in the bushy cells. Taken together, under our experimental conditions, we observe neuronal TeNT-dependent cell death in the VCN that is not accompanied by a loss of calyces in the contralateral MNTB, suggesting that GBCs are rather insensitive to TeNT-expression. However, we cannot formally exclude that a minor fraction of calyces is indeed lost upon prolonged TeNT expression. Nevertheless, our conclusions on the remaining calyces remain unaffected by this potential loss of a few calyces.

TeNT affects synaptic transmission within minutes, when directly applied to the presynapse via a patch pipette at high concentrations (Sakaba et al., 2005). However, prolonged expression is required under our experimental conditions due to the comparatively slow build-up of efficient TeNT concentrations at the synapse, the sheer amounts of VAMP2 copies that needed to be cleaved at the calyx (∼70 copies/SV, ∼70,000 SVs/calyx, Takamori et al., 2006; Sätzler et al., 2002, respectively) and the need to accumulate sufficient amounts of fluorescently labeled TeNT to be detected in acute slices. Effective synaptobrevin cleavage by TeNT after 14 days of *in vivo* incubation was shown by electrophysiology. TeNT expression drastically reduced the frequency of sEPSCs in MNTB principal neurons. However, we still detected some remaining sEPSCs in MNTB cells contacted by TeNT expressing calyces. These sEPSCs can either represent spontaneous release from non-calyceal inputs, or they originate from the calyx, despite TeNT expression. Excitatory inputs to MNTB neurons independent of the calyx of Held have been demonstrated both structurally and functionally (Holcomb et al., 2013; Hamann et al., 2003, respectively). Evoked EPSCs from these inputs have been shown to be smaller in amplitude and about three times slower in rise time than calyceal evoked EPSCs (Hamann et al., 2003). The properties of spontaneous EPSCs from those inputs are not known but it can be assumed that their rise time is somewhat slower than that of calyceal sEPSCs, too. Although we found a tendency toward such a slowing (Figures 4B, E) this trend did reach significance, suggesting that non-calyceal inputs may represent a higher portion of sEPSCs in MNTB neurons contacted by TeNT-expressing calyces as compared to controls. The origin of the non-calyceal inputs is unknown, but it is highly unlikely that they originate from the contralateral VCN (Kandler and Friauf, 1993; Borst and Soria van Hoeve, 2012) or express Math5 (Saul et al., 2008). Therefore, neurotransmitter release from these terminals is not affected in our experimental paradigm. Alternatively, the remaining sEPSCs could still originate from the calyx of Held, despite the expression of TeNT, in line with the knock-out of VAMP2 not abolishing synaptic release completely (Schoch et al., 2001, Deák et al., 2004). However, the knock-out of VAMP2 leads to the upregulation of VAMP1, resulting in a partial functional compensation (Imig et al., 2014). Under our experimental conditions, an upregulation of VAMP1 is unlikely to account for the remaining sEPSCs, as TeNT also cleaves VAMP1 in mice (Humeau et al., 2000). Nevertheless, sEPSC could be mediated by other v-SNAREs such as VAMP4, VAMP7 and Vti1a that are insensitive to TeNT and could thus compensate for the loss of VAMP1 and VAMP2. Spontaneous SV release depending on one of these v-SNAREs has not yet been reported at the calyx of Held, but in various other synapses including the neuromuscular junction and bouton-type synapses in hippocampal cultured neurons (Hua et al., 2011; Raingo et al., 2012; Ramirez et al., 2012; Bal et al., 2013; Liu et al., 2019). It is thus conceivable that the remaining sEPSCs recorded in MNTB neurons contacted by TeNT-expressing calyces represent a mixture of non-calyceal inputs and calyceal sEPSCs mediated by TeNT-insensitive v-SNAREs.

Despite some residual sEPSCs recorded in MNTB neurons contacted by TeNT-expressing calyces, we are confident that neurotransmitter release from the calyx is indeed virtually abolished by TeNT for two reasons. First, we were unable to electrically induce synchronous or asynchronous neurotransmitter release from TeNT calyces, not even by trains of high frequency stimulation, although this was possible in all WT synapses tested. Second, our EM results show a close to complete lack of SVs in close proximity to the AZ in TeNT-expressing calyces. Since these membrane-near SVs constitute the ready releasable pool (Imig et al., 2014), we conclude, in accordance with our electrophysiological results, that TeNT-expressing calyces are basically devoid of release-ready SVs and thus - most likely - practically silent. Of note, we still observed rather small, slow inward currents in some of the TeNT-expressing calyces (e.g., Figure 5A inset). Such a slow SV release that is apparently insensitive to acutely applied TeNT has been described at the neuromuscular junction and calyx of Held (Dreyer and Schmitt, 1983; Bevan and Wendon, 1984; Sakaba et al., 2005). At the calyx of Held, upon acute application of TeNT via a patch pipette a slow component of SV release has been shown to persist for 9 min in response to prolonged presynaptic depolarization (Sakaba et al., 2005). The slow inward currents obtained here may thus represent a slow component release employing TeNT-insensitive SVs which would involve an alternative v-SNARE protein (see above), although they have not been implicated in evoked SV release, yet. However, it also seems rather unlikely that the slow inward current is carried by SVs that rely on VAMP1 or 2, for two reasons: first, the published acute TeNT application is limited to minutes, 9 min in the case of the calyx, while we express TeNT for 14 days prior to recordings. It seems unlikely that a fraction of VAMP1 or 2 molecules is guarded from TeNT by an assembled SNARE complex for such extended periods. Second, prolonged presynaptic depolarizations result in far higher presynaptic calcium concentrations than those induced by trains of action potentials, which did not result in synchronous or asynchronous SV release in TeNT-expressing calyces (Figures 5C, D). Prolonged depolarization thus induces the release of SVs that are not available upon action potential-induced SV fusion and more release reluctant, although they possess an at least partially assembled SNARE complex (Sakaba, 2006).

Interestingly, we not only observed a marked decrease in SVs in the immediate vicinity of the presynaptic plasma membrane, but in AZ-associated SVs in general. The numbers of SVs were reduced for ∼100 nm into the presynaptic volume. This could be due to two reasons: a deficiency in local SV recycling or the impairment of SV supply from the soma. VAMP2 has been reported to be involved in synaptic endocytosis at the calyx of Held and hippocampal synapses (Xu et al., 2013; Zhang et al., 2013, respectively). However, newly supplied SVs are of course equipped with VAMP2, too. Since TeNT is not specifically localized to the calyx, but broadly expressed throughout the bushy cell soma, it is conceivable that the entire VAMP2 content of the newly generated SVs is cleaved before the arrival at the calyx, therefore not functional, and thus marked for degradation. We consider the former case more likely for two reasons: first, we should have observed an accumulation of newly supplied, release-incompetent SVs associated with the AZ, which we did not. Second, we observed an increase in synapse volume in TeNT-expressing calyces (Figure 6), which could indicate an impairment in membrane handling in these synapses, although the synaptic surface area was unchanged. The clarification of the exact mechanism causing the observed decrease in SV number provides an interesting topic for future studies.

### Synaptic silence does not affect synapse maturation or the survival of MNTB neurons

Silencing of synaptic transmission by abolishing neurotransmitter release has been achieved by a number of genetic manipulations, including the knock-out of essential presynaptic proteins, without precluding synaptogenesis (Verhage et al., 2000; Varoqueaux et al., 2002; Deák et al., 2004). Formation of the calyx of Held occurs before E17 as a bouton-shaped contact (Marrs and Spirou, 2012) that expands to its juvenile, cup-shaped form between P4 and P6, concomitant with the establishment of a one-to-one connectivity (Hoffpauir et al., 2006; Holcomb et al., 2013). The mature, fenestrated shape of the calyx is reached by P14-16, shortly after the onset of hearing (Kandler and Friauf, 1993; Ford et al., 2009, Borst and Soria van Hoeve, 2012). We only injected the TeNT-encoding AAVs at P6/7, at a time when the calyx has reached the juvenile stage. Our experimental conditions thus do not interfere with initial synapse formation, synapse expansion and synaptic pruning. However, TeNT was already expressed at the start of fenestration, although only for ∼4 days. Since this expression time is short, and TeNT may not have reached effective concentrations, yet, it is difficult to draw conclusions on whether the start of the fenestration process depends on synaptic activity. Nevertheless, fenestration is only close to complete by P14, 8 days after the start of TeNT expression. At this time, it is likely that a considerable amount of TeNT is present at the synapse and it is thus tempting to speculate that fenestration proceeds even under conditions of reduced or abolished neurotransmitter release. This is surprising because fenestration has been suggested to assist in efficient glutamate clearance from the synaptic cleft (Renden et al., 2005) and thus represent a morphological adjustment necessary to sustain the high frequency synaptic activity of the mature calyx (Crins et al., 2011; Sonntag et al., 2011). Notably, unspecific silencing of the calyx by unilateral denervation even resulted in a more complex fenestration of the calyx (Grande et al., 2014), while reducing synaptic activity by ear occlusion leads to smaller endbulbs of Held in the VCN (Zhuang et al., 2017; Wong and Xu-Friedman, 2022). In the present study, we did not observe differences in synapse size or complexity. This apparent discrepancy may be due to the broad impairment of the auditory system by denervation and the fact that synapses are still capable of adjusting neurotransmitter release under both of these experimental conditions.

The lack of neurotransmitter release from inner hair cells in mice lacking the L-type channel Ca_v_1.3 has been shown to result in pronounced cell death throughout the auditory brainstem (Hirtz et al., 2011; Satheesh et al., 2012). However, although Ca_v_1.3 is highly expressed in auditory brainstem neurons, the lack of it does not abolish neurotransmitter release, but changes action potential properties (Hirtz et al., 2011). Therefore, we consider it likely that the observed loss of neurons in Ca_v_1.3 knock-out mice is not due to the lack of neurotransmitter release, but rather impaired calcium signaling pathways at the soma. Under our experimental conditions, we neither observed a loss of calyces from the MNTB, nor a decrease in MNTB principal neurons. This allowed us to specifically examine the maintenance of the calyx of Held in the absence of calyceal neurotransmitter release.

### Synaptic maintenance of the calyx of Held is largely independent of neurotransmitter release

TeNT expression has been performed in various regions of the central nervous system with variable results, ranging from the failure to establish specific synaptic connections (Kerschensteiner et al., 2009) via defects in synaptic refinement and pruning (Yu et al., 2004; Wang et al., 2007; Lopez et al., 2012) to the loss of a fraction of synapses with no further impact on the remaining ones (Sando et al., 2017; Sigler et al., 2017; Quinn et al., 2019). Under our experimental conditions, we do not interfere with synaptic formation, refinement or pruning, since these events take place before TeNT reaches an effective concentration at the calyx (see above). However, we found only a minor morphological adaptation of the mature calyx of Held to the absence of synaptic activity with a thickening of the calyx being the sole significant effect. Interestingly, we did not observe a reduction in AZ number that would resemble the loss of synapses reported after silencing of the forebrain or neuronal cultures (Sando et al., 2017; Sigler et al., 2017; Quinn et al., 2019). This may be explained by the finding that TeNT expression mainly interferes with the formation of synapses in cultured neurons (Quinn et al., 2019), a process that is probably completed before TeNT expression becomes effective in our study (Borst and Soria van Hoeve, 2012). Additionally, and perhaps even more surprisingly, we neither observed a significant difference in the number or size of postsynaptic AMPA receptor clusters nor in their subtypes in silent synapses. MNTB neurons contacted by TeNT-expressing, silent calyces only tended to incorporate more of the juvenile GluA1 subunit into their synapses, which is particularly surprising, since the mature calyx of Held reaches synaptic transmission rates of up to ∼350 Hz during sound perception (Kopp-Scheinpflug et al., 2008) and its silencing thus induces a particularly drastic decrease in synaptic activity. Further experiments will be needed to investigate if the high frequency auditory brainstem synapses are generally maintained during synaptic silence or if this applies only to the calyx and possibly other giant synapses.

### Limitations

Although the data presented here imply that the expression of TeNT and the resulting absence of synaptic transmission does not result in the degradation of the calyx of Held but has only minor effects on synapse morphology, our study has certain limitations. Most importantly, we still recorded some few sEPSCs at the MNTB principal cell after 14 days of TeNT expression that are of unknown origin but can result from calyceal SV release mediated by TeNT-insensitive v-SNAREs (see above). We can also not formally exclude that the remaining sEPSCs are due to a misalignment of the pre- and postsynaptic compartments. SV fusion outside the AZ would result in prolonged diffusion times till the glutamate reaches the receptors and thus slower rise times (see Budisantoso et al., 2013 for details on glutamate diffusion kinetics). However, neither our immunohistochemical data nor our ultrastructural analysis show evidence for a misalignment of the synaptic compartments. A technical limitation of our study is the direct stimulation of TeNT-expressing axons giving rise to the calyx of Held in the terminal’s immediate vicinity. We cannot guarantee that TeNT-expression does not hamper the efficient invasion of the AP into the calyx, although we are not aware of any reports or mechanisms that would suggest such an effect of TeNT. Additionally, our study does not determine the exact timepoint at which the calyx is silent. The determination of this is hampered by the fact that we require approximately 10 days of expression to accumulate enough fluorescent protein at the calyx to identify transduced synapses, under our experimental conditions (Vasileva et al., 2012; Körber et al., 2015).

## Conclusion

Taken together, our results show that synaptic activity of the calyx of Held is dispensable for maintenance and supposedly also for the later stages of synaptic maturation. The calyx may thus be regarded essential for the system and therefore maintained independent of synaptic activity. Interestingly, we did not even observe changes in the number of AZs or in the postsynaptic AMPA receptor composition. The functional importance of the calyx in the sound source localization circuit is evident by its specialized morphology and the presence of several hundreds of AZs (Joris and Trussell, 2018). It thus seems that the brain maintains such a central relay station in an activity-independent, hardwired and probably genetically encoded manner. Future studies, using a more broadly, systemic expression of TeNT in the auditory brainstem, may provide further insights into the mechanisms of synaptic maintenance in the absence of synaptic transmission in this high frequency signaling brain area.

## Data Availability

The raw data supporting the conclusions of this article will be made available by the authors, without undue reservation.
